# Therapeutical Notes

**Published:** 1899-03-04

**Authors:** 


### THERAPEUTICAL NOTES.

### Treatment of Graves' Disease.

Hector Mackenzie (*Amer. Journal of Med. Sciences*) advises that one or two drachms of fresh thymus gland or its equivalent in the dried forms should be given daily to improve the general condition of the patient, though it has no specific action on the disease. Deguy (*Clinical Journal*, October 5th) recommends small doses of digitalis in some cases where the heart is greatly disturbed in rhythm and power. If there is much cardiac pain nitro-glycerine is useful, and for palpitations an ice-bag or cold compresses may be applied to the precordium, or a spray of ether or ethyl chloride may be used.

### Gastralgia.

The *Practitioner* of November gives the following formula recommended by Ewald, of Berlin, for the relief of certain forms of gastric pain:—

℞ Codeinæ phosphatis, gr. ¼.

Bismuthi subnitratis, gr. 5.

Sacchari Lactis, gr. 3.

To make one powder, to be taken every two hours.

Hale White gives an admirable prescription for flatulence and pain, as follows:—

℞ Sp. Cajuputi,
Sp. Chloroformi,
Sp. Am. Aromatici āā p. æquales.

M. et signe. A teaspoonful to be taken in water occasionally.

